# 3D Analytic Cone-Beam Reconstruction for Multiaxial CT Acquisitions

**DOI:** 10.1155/2009/538389

**Published:** 2009-08-30

**Authors:** Zhye Yin, Bruno De Man, Jed Pack

**Affiliations:** CT and Xray systems and applications laboratory, GE Global Research, Niskayuna, NY 12309, USA

## Abstract

A conventional 3rd generation Computed Tomography (CT) system with a single circular source trajectory is limited in terms of longitudinal scan coverage since extending the scan coverage beyond 40 mm results in significant cone-beam artifacts. A multiaxial CT acquisition is achieved by combining multiple sequential 3rd generation axial scans or by performing a single axial multisource CT scan with multiple longitudinally offset sources. Data from multiple axial scans or multiple sources provide complementary information. For full-scan acquisitions, we present a window-based 3D analytic cone-beam reconstruction algorithm by tessellating data from neighboring axial datasets. We also show that multi-axial CT acquisition can extend the axial scan coverage while minimizing cone-beam artifacts. For half-scan acquisitions, one cannot take advantage of conjugate rays. We propose a cone-angle dependent weighting approach to combine multi-axial half-scan data. We compute the relative contribution from each axial dataset to each voxel based on the X-ray beam collimation, the respective cone-angles, and the spacing between the axial scans. We present numerical experiments to demonstrate that the proposed techniques successfully reduce cone-beam artifacts at very large volumetric coverage.

## 1. Introduction

Since 1990, multislice or multidetector-row computed tomography (CT) systems have become the standard CT architecture for premium medical scanners: the detector has multiple rows, that is, a 2-dimensional array of detector cells, yielding a cone-beam geometry. Since CT systems with this geometry do not generate the rays to be perpendicular to the rotational axis, 2D image reconstruction algorithms result in reconstructions that suffer from cone-beam artifacts. For axial scan mode where the table does not move during gantry rotation, Feldkamp, Davis, and Kress proposed a 3D cone-beam reconstruction algorithm (FDK) that is an adaptation of the 2D fan-beam filtered backprojection (FBP) to a cone-beam geometry [1]. The Feldkamp algorithm works well near the mid-plane and near the center of the rotation, but artifacts occur and get worse as coverage increases. The data are fundamentally incomplete in 3D axial scans with limited detector size. Therefore, with the presence of perturbation, the reconstruction of outer slices will always suffer from cone-beam artifacts, regardless of the reconstruction approach [2]. 

In helical cone-beam scans, the data are fundamentally complete, provided that the helical pitch is not too high. Therefore, exact reconstruction can be achieved [3–5]. Katsevitch was the first to propose an exact 3D filtered backprojection algorithm for helical cone-beam reconstruction, which consists of filtering along special lines on the detector followed by backprojection [3]. The FDK algorithm has also been adapted for helical scan modes, resulting in nonexact or approximate reconstruction [6, 7]. 

Other interesting reconstruction problems have been posed by the demand for dynamic object imaging, such as cardiac CT. Theoretically, a 2D object can be reconstructed accurately if all its line integrals are measured at least once. This condition leads to the notion of half-scan or short-scan, which means that the scan interval per acquisition is only 180° plus the fan angle. Various weighted FDK-types of algorithms are available for half-scan case [8, 9]. Furthermore, acquisitions with an even shorter scan interval, so-called super short scan, have gained popularity since the dynamic organs can be restricted to a limited region of interest (ROI) in some clinical applications. A smaller ROI can be exactly reconstructed with a shorter scan interval [10]. 

In axial scan mode, conventional 3rd generation CT systems suffer from increased cone-beam artifacts with increasing coverage, due to incomplete data and suboptimal processing of the available data. The efforts to increase axial scan coverage without sacrificing image quality led to the weight-based cone-beam reconstruction algorithms for single circular trajectory acquisitions [11, 12] as well as sequential scanning algorithms to combine multiple axial acquisitions sequentially taken by a conventional 3rd generation CT system [13, 14]. With multiple axial acquisitions, either sequentially acquired with a conventional 3rd-generation CT system or simultaneously acquired with a multisource CT system with multiple longitudinally offset sources [15], a more complete dataset can be acquired, resulting in reduced cone-beam artifacts and increased scan coverage. The longitudinal truncation problem from a single axial CT acquisition can be solved by the additional axial acquisition with offset, providing conjugate rays which are not available for the single CT acquisition. In other words, the tear drop shape of Radon frequency is missing in a single axial CT acquisition due to the wide cone angle and longitudinal truncation. Data from additional offset axial scans will provide some of missing Radon frequency and further reduce cone-beam artifact. To exploit this additional information, novel 3D cone-beam reconstruction algorithms are proposed and are discussed in detail in this paper. 

In Section 2, the possible system architectures for multi-axial acquisition CT are described. In Sections 3 and 4, we describe geometries and algorithms for multi-axial acquisitions for full-scan and half-scan mode, respectively. In Section 5, we present experimental results comparing the proposed multi-axial acquisition and reconstruction to conventional 3rd generation CT acquisition and reconstruction. Section 6 summarizes the main conclusion.

## 2. Multiaxial CT Acquisitions

In this section, we briefly discuss three possible multi-axial CT acquisition system architecture concepts and the corresponding full-scan and half-scan acquisition and reconstruction modes. 

### 2.1. Multiaxial Acquisition System Architectures

**Figure 1:**
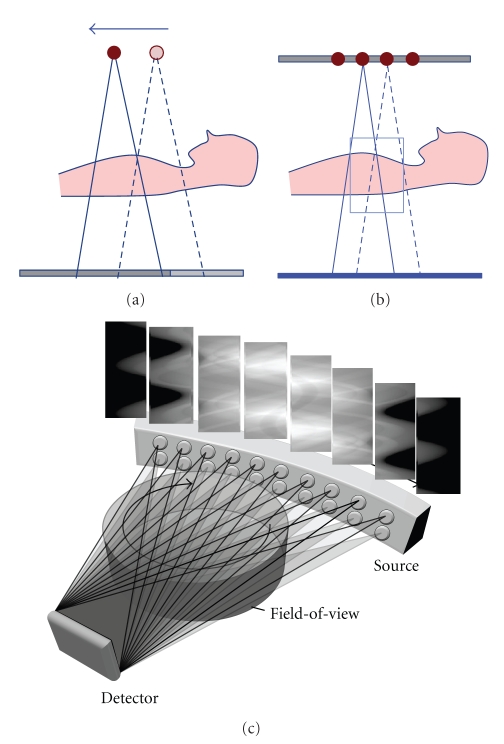
Multi-axial acquisition system concepts: (a) multiple sequential axial scans taken by single source 3rd generation system, (b) a system with multiple sources distributed longitudinally, and (c) a multisource inverse geometry source (MS-IGCT) with 2 by 10 area source. Each subsinogram can be rebinned to conventional 3rd generation sinogram.

First, the simplest way to acquire multi-axial data without modifying any system hardware is to take a series of axial scans sequentially (Figure 1(a)). After a first axial scan, the table is moved to the next desired position, where a second scan is performed with some overlap with the previously scanned region. The table displacement is equal to or smaller than the single axial scans coverage at the isocenter to prevent gaps between consecutive scans. Since each axial scan is acquired at a different time frame, one challenge is to seamlessly combine those datasets and avoid any artifacts due to temporal misregistration, particularly in dynamic applications. 

Second, a multi-source system with longitudinally distributed sources or a line-source CT is another way to acquire multiple axial data. Two or more focal spots are distributed along the z-axis and are alternatively emitting X-ray. There is no spatiotemporal misregistration between the two (or more) axial datasets, which is preferable for dynamic applications (Figure 1(b)). 

Third, a multi-source inverse geometry CT (MS-IGCT) consists of a number of focal spots distributed in x (trans-axially), each emitting a relatively narrow X-ray beam through a small portion of the field-of-view, shown in Figure 1(c). After a full 360° rotation, the equivalent of a full 3rd generation dataset is acquired after rebinning. The nature of the distributed source also makes it easy to use multiple series of focal spots distributed longitudinally, which offers another way to perform a multi-axial acquisition and therefore provide increased volumetric coverage and reduced cone-beam artifacts [15]. After xy rebinning, a multi-source IGCT dataset can be treated as a line-source CT acquisition [16]. 

In this paper, we present analytic cone-beam reconstruction algorithms for multi-axial acquisitions. While we focus on the line source CT case, the methods and results translate directly to any other type of multi-axial acquisition.

### 2.2. Scan and Reconstruction Mode

**Figure 2:**
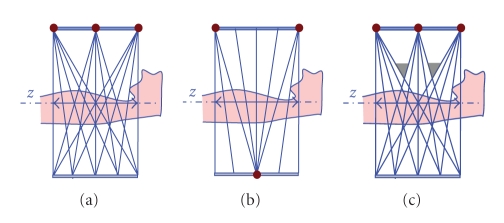
Multi-axial acquisition geometries with 3 longitudinal acquisitions (source positions marked as red dots): (a) fully opened collimation, (b) full-scan mode with semi-closed collimation (central source is shown in opposite side.), and (c) half-scan mode with fully opened collimation.

The multi-axial acquisition architectures described in the previous section has two scan and reconstruction modes to meet various clinical needs: (1) a full-scan mode, for which we will propose a window-based reconstruction and (2) a half-scan mode, for which we will propose a cone-angle-dependent weighting reconstruction. Figure 2 shows the transaxial geometry for these two scan modes. Note that the longitudinal coverage of the architecture in Figure 2 is equal to the detector height. 

The most straightforward way to combine data from multi-axial acquisitions is to reconstruct the volumes corresponding to each single axial acquisition separately and computing a weighted combination of the reconstructed slices from the different axial datasets. In practice, most of slices will be substituted with the slices reconstructed from the single axial dataset at same *z*-location (binary weights), but there will be some feathering in the weights to gradually make a transition from one axial reconstruction to the next. This configuration requires a relatively large detector illumination to ensure overlap between the consecutive reconstructed volumes Figure 2(a), and it leads to nonuniform dose and noise profiles in each coronal and sagittal slice, described in more detail later. In Section 3, we present an improved, window-based reconstruction algorithm for multi-axial full-scan acquisitions.

In half-scan mode, conjugate rays are in general not available. However, just like for full-scan, the desired volume can be reconstructed by taking a weighted combination of the reconstructions from each axial dataset. Again, a sufficiently large detector illumination is required to avoid any gap in the volumetric coverage, as shown in Figure 2(c). In Section 4, we present an improved cone-angle-based weighting approach for multi-axial half-scan reconstruction.

### 2.3. Dose Efficiency

The multi-axial CT architecture introduces additional X-ray sources or scans, and the concerns on additional X-ray dosage should be addressed. Instead of discussing absolute X-ray dose which can be traded off with the image quality, we would like to focus on the dose-efficiency to utilize the best out of the given X-ray dosage. To achieve the maximum dose efficiency, first every X-ray from the tube should be detected. The multi-axial CT architectures described in previous section with big enough detector will certainly meet this criterion. 

**Figure 3:**
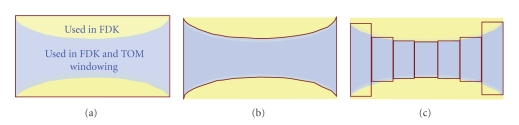
The detector utilization of the FDK-based and TOM windowing reconstruction approaches. The area closed by red lines represents the actual acquired area of the detector for (a) multi-axial scan/line source CT, (b) line source CT curved collimator, and (c) multi-source inverse geometry CT. The area in blue represents the data required to reconstruct volume using TOM windowing approach. Note that TOM windowing has an advantage over FDK in detector utilization if data is acquired by the line-source CT with the collimator or the multi-source inverse geometry CT.

Second, every detected projection should be used in the image reconstruction process. In other words, we can achieve the maximum dose efficiency by only acquiring the projection data needed in the reconstruction process. The detector utilization of the multi-axial CT acquisitions and reconstruction algorithms are shown in Figure 3. For multi-axial scan/line-source CT, shown in Figure 3(a), the data from whole detector are acquired. Note that if TOM windowing is the choice of the reconstruction, the detector utilization is not good since the area in yellow area get thrown away. For line-source CT with curved collimator, shown in Figure 3(b), TOM windowing approach can achieve the maximum dose efficiency by only acquiring the projection data needed. For multi-source inverse geometry CT, shown in Figure 3(c), subsinograms from transaxially distributed sources are well matched with the area needed for the TOM windowing reconstruction approach, and therefore TOM windowing approach can achieve the maximum detector utilization. 

**Figure 4:**
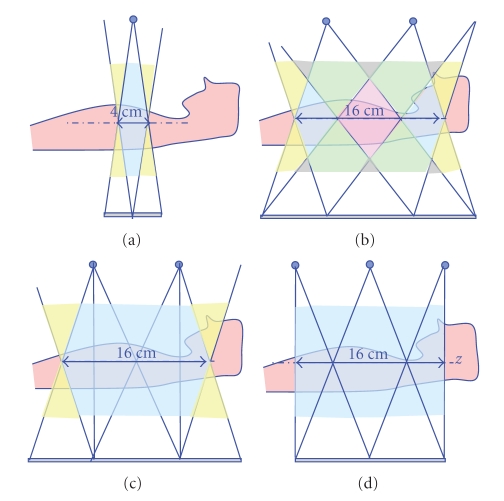
The flux uniformity of the various CT architectures and associated reconstruction approaches: (a) 3rd generation CT with FDK reconstruction, (b) line source CT with FDK slice substitution approach, (c) line source CT with TOM windowing reconstruction, and (d) multi-source inverse geometry CT with TOM windowing approach. The areas with different flux level are shown in different colors.

Finally, a uniform flux profile should be achieved along the reconstruction volume to maximize dose efficiency since the nonuniform flux/noise profile degrades the dose-efficiency. The flux uniformity of the various CT architectures and associated reconstruction approaches are shown in Figure 4. For line-source CT with FDK slice substitution approach, shown in Figure 4(b), the region marked in pink get twice more X-ray flux than the region marked in blue. This flux nonuniformity can be mitigated by using TOM windowing approach instead, shown in Figure 4(c).

## 3. Window-Based Cone-Beam Reconstruction for Full-Scan Mode

In helical reconstruction algorithms, a so-called Tam-window is used, named after one of the inventors, Kwok Tam [17], and defined as the projection of the helical source trajectory on the detector. Only the portion of the physical detector confined by the Tam-boundaries is used to reconstruct the desired volume. We extend this concept to multiple circle acquisitions that are offset in the *z*-direction. We project the circular source trajectories on the detector and for convenience label this the Trajectory Opposite Mapping window (TOM). A similar idea was explored in [13] to determine the optimal distance between sequentially acquired datasets and to reconstruct those datasets. Unlike [13], the approach presented here does not include rebinning to a rectangular virtual detector but is based on explicit windowing during backprojection. The windowing concept is based on the idea that we can patch rays from one source with conjugate rays from a longitudinally adjacent source after about half a rotation (180°), in order to jointly cover the object completely. The TOM window is chosen such that there is no overlap between two longitudinally adjacent sources where one is apart from the other by 180° as illustrated in Figure 2(b). This leads to a tessellation during backprojection that is similar to the one achieved with Tam-windowing for helical acquisitions but in vertical planes.

**Figure 5:**
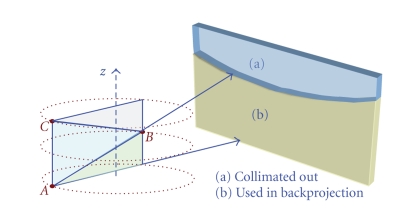
Efficient TOM windowing: during the backprojection of each voxel and each view, it is decided if the projection of that voxel falls inside the TOM window and if not, the contribution from that view is discarded. 3 longitudinal sources are marked as *A*, *B*, and *C*.

One way to use the tessellation property is to synthesize plane integrals. First the Radon transform of the projections is computed, and the derivatives are taken to apply Grangeat's theorem. Tam's tessellation approach is then used to patch the triangular regions. Addition of a boundary term is required for more accurate (but still nonexact) reconstruction. Finally the desired volume can be reconstructed by taking a 3D inverse Radon transform. Note that not all plane integrals can be computed due to the nature of the multi-axial architecture, but missing integrals can be estimated by interpolation. A second way to apply the tessellation approach is using filtered backprojection, which is our preferred approach. We apply an existing filtered backprojection technique, such as FDK, and we combine it with a TOM-windowed backprojection. Geometrically, the cones from complimentary sources can match perfectly with each other so that data between the TOM window boundaries jointly cover the entire volume. This means that rays from adjacent sources should intersect each other at the isocenter, as shown in Figure 2(b). This collimation gives us several benefits: all sources have the same worst-case cone-angle, the X-ray flux is uniform throughout the patient, and correspondingly the image noise is relatively uniform. We now assume 3 source spots distributed along the *z*-axis for simplicity, but the same principle can be applied to the case with two or more than 3 source spots. For the center source spot, two axial trajectories from top and bottom source can be projected onto the physical detector. These projected points will form TOM-boundaries which bound the top and bottom of the detector. We have 2 areas to be considered. The first area, (a), in Figure 5, includes the regions above and below the TOM-boundary. The second area, (b), is the region between the two TOM-boundaries. When reconstructing a voxel (or any point in the image volume) whose projection onto the detector is in region (a), we discard that particular view (i.e., we do not apply the backprojection of that view to that voxel), because data from the top source will be used there instead (and presumably also provide better or more complete information for that particular angle). 

**Figure 6:**
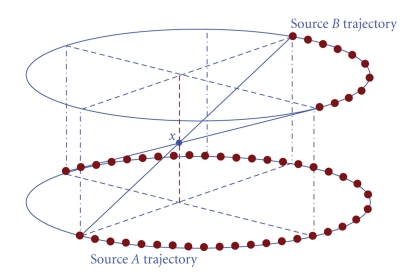
View contributions from source *A* and source *B* to a given reconstruction point *x*.

The windowing can be achieved by applying a binary mask to the filtered projection data, but this approach suffers from quantization artifacts. A better way to implement the TOM window is to make a decision during the backprojection of each voxel for each view, by determining whether or not the voxel analytically projects inside the TOM window. This implementation has less quantization error but higher computational complexity. Figure 6 illustrates how some views contribute to a voxel and others do not in a window-based backprojection. 

The detector region surrounded by the TOM boundaries for a given source is selected by a weight function $\;w(\hat{\gamma },\hat{\alpha })$. The weight function $w(\hat{\gamma },\hat{\alpha })$ ensures that only the set of voxels projected onto the detector between the TOM boundaries are getting a nonzero contribution during backprojection. Therefore

(1) $$w\left(\hat{\gamma },\hat{\alpha }\right)=\{\begin{array}{cc} 1, & \gamma_{\text{bottom}}\left(\hat{\alpha },i\right)\leq \hat{\gamma }\leq \gamma_{\text{top}}\left(\hat{\alpha },i\right),\\ 0, & \text{otherwise}, \end{array}$$

where $\gamma_{\text{bottom}}\left(\hat{\alpha },i\right)$ is the bottom boundary of the TOM window, which corresponds to the projection of the (*i* − 1)th source trajectory, and $\gamma_{\text{top}}\left(\hat{\alpha },i\right)$ is the top of the TOM window, which corresponds to the projection of the (*i* + 1)th source trajectory on the physical detector. Depending on the geometry of multiple longitudinal sources, these boundaries can be computed up front or during backprojection. If the source-to-isodistances (SIDs) for all sources are identical, the corresponding boundaries are

(2) $$\begin{array}{c} \gamma_{\text{bottom}}\left(\hat{\alpha },\text{i}\right)=z_{s}\left(i\right)-\frac{d_{\text{source}}\cdot \text{SDD}}{2\cos\;\;\hat{\alpha }\cdot \text{SID}}, \end{array}$$

(3) $$\begin{array}{c} \gamma_{\text{top}}\left(\hat{\alpha },\text{i}\right)=z_{s}\left(i\right)+\frac{d_{\text{source}}\cdot \text{SDD}}{2\cos\;\;\hat{\alpha }\cdot \text{SID}}, \end{array}$$

where SDD means source-to-detector distance, *d*_source_ is denoted as the longitudinal spacing between 2 adjacent sources, and *z*_*s*_(*i*) is the longitudinal coordinate of the *i*th source. 

As described above, TOM windowing is implemented during the backprojection step for each longitudinally located source. The backprojected volume associated with a given source will be partial, and the reconstruction step will be completed with the summation of all partial volumes. Thus, the final reconstruction is given by

(4) $$\hat{f}\left(\underline{x}\right)=\overset{N}{\underset{i=1}{\sum }}{\hat{f}}_{i}\left(\underline{x}\right),$$

where *N* is the total number of longitudinally offset sources, and ${\hat{f}}_{i}(\underline{x})$ is the partial reconstruction volume associated with the ith longitudinally located source. 

The FDK algorithm employs a ramp filter in its filtering step. This works well for a full axial scan because the boundary terms incurred by the difference between a fan-beam geometry and a parallel-beam geometry are canceled out by backprojecting over a full rotation [18]. However, when disjoint source path segments are combined through TOM windowing, the use of a ramp filter is no longer valid; that is, the boundary terms are not canceled out. These undesired terms can be avoided by replacing a ramp filter with the combination of a parallel derivative, also called a view dependent derivative, and a Hilbert transform. Parallel derivatives are computed by combining the derivative along a detector column with the derivative in the source coordinate. The Hilbert transform is applied to the derivative data [3]. Therefore *i*th partial reconstruction volume is given by

(5) $$\begin{array}{c} {\hat{f}}_{i}\left(\underline{x}\right)=\frac{1}{2\pi }\int_{\Lambda }\frac{w\left(\hat{\gamma },\hat{\alpha }\right)}{|\underline{x}-\underline{a}\left(\lambda ,i\right)|}\cdot \frac{d}{ds}{\left[b\left(s,i,\underline{\theta }\right)\right]|}_{s=\lambda }*h\left(\sin\;\alpha \right)d\lambda \\ b\left(\lambda ,i,\underline{\theta }\right)=p\left(\lambda ,\alpha ,\gamma ,i\right), \end{array}$$

where *h*(sin *α*) is the Hilbert kernel in spatial domain, and $\underline{\theta }$ is a unit vector pointing from a source location *a*(*λ*) to the reconstructed voxel *x*, representing the detector fan angle and cone angle for a given voxel *x*, source *i*, and source location *a*(*λ*, *i*). Equation (5) can be seen as a special approximate case of Kasevich's general formula where filtering step is reduced to 1D convolution along the detector rows [3].

The view derivative can be computed at interlaced sampling locations, as described for a helical trajectory in [4, 19], by noting that an axial scan is simply a helical scan with the helical pitch set to zero. The Hilbert transform can be applied with a half pixel shift in the detector column direction also described in [4]. This is done by convolving the differentiated sinogram with the kernel of the Hilbert transform and assigning the result to sample positions that are offset by one half of a pixel along the column direction. This approach makes it possible to use the TOM windowing approach without introducing artifacts as a result of the discontinuous view weights with ramp-filter-based approaches. 

**Figure 7:**
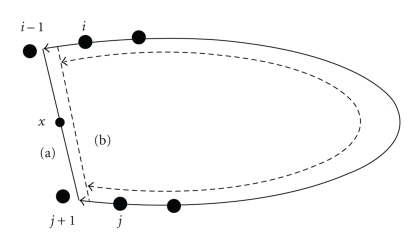
Discrete view sampling: true pi-line, (a) in solid line and approximated pi-line, (b) in dashed line, due to the discrete view sampling. Note that pi-segment associated with (b) is now smaller.

Since only a finite number of discrete view samples will be taken during real scanning, the binary windowing operation can introduce artifacts, which are also discussed in [4]. To see this, consider the reconstruction point *x*, shown in Figure 7. The pi-line corresponding to a given point *x* is shown in solid line, and the corresponding pi-segment is shown in solid line with bidirectional arrow. The end points of the pi-line are closer to the pair of source samples just outside the pi-segment (i.e., (*i* − 1) and (*j* + 1)) than they are to the pair just inside the pi-segment (i.e., (*i*) and (*j*)). However, application of discrete TOM windowing rejects the view samples at (*i* − 1) and (*j* + 1) completely. This effectively approximates the pi-line as being the dashed line in Figure 7, and the corresponding pi-segment is shown in a dashed line with bidirectional arrow. 

This problem can be mitigated by an additional linear smoothing step. Instead of taking binary weights near the TOM boundaries, partial weights are determined by how close projected voxels are to the boundary. One could define a fixed width region along the TOM boundary, and whenever voxels are projected into that region, they will receive a partial back-projection contribution instead of a full contribution. However this approach will not result in desired smoothing effect because the weight at a certain source position and the weight at the corresponding conjugate source position might not add up to one. Instead, we define a fixed longitudinal interval around each voxel and project (the endpoints of) this interval onto the detector. This way the conjugate weights contributing to a given voxel will add up to one.

The TOM windowing approach described above combines contributions from adjacent source positions. However, it can be extended to combine contributions from source positions that are not immediately adjacent. 

## 4. Cone-Angle-Weighted Cone-Beam Reconstruction for Half-Scan Mode

In half-scan mode, triangular patching of conjugate data from longitudinally offset axial scans is no longer possible. Combining data from multiple longitudinally offset axial scans becomes challenging because the reconstruction volume is now divided into several regions: regions with no illumination and regions illuminated once, twice, or three times, which means that complementary information is not always available. Various weighting approaches utilizing complementary information have been proposed by introducing the scale factors representing how much information each projection contributes to a given voxel [12]. 

**Figure 8:**
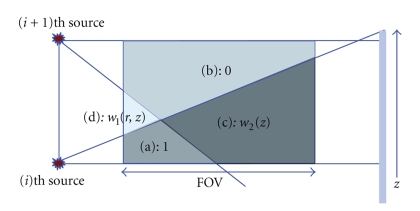
Multi-source half-scan regions at a given fan angle: (a) a region illuminated by only (*i*)th source, (b) a region illuminated by only (*i* + 1)th source, (c) a region illuminated by both (*i*)th and (*i* + 1)th sources, and (d) a region without illumination.

We propose a new approach where the weights are computed based on the cone-angle on a voxel and per view basis. Figure 8 shows two sources and their corresponding X-ray beams. For a given fan angle *α*(*λ*, *x*), a reconstructed voxel is denoted as *x*(*r*, *z*), where *r* is defined as the distance from a given source to a given voxel in the *xy*-plane, and *z* is the longitudinal coordinate of a given voxel. A voxel belongs to one of 4 regions in Figure 8, (a) a region illuminated by only the (*i*)th source, (b) a region illuminated by only the (*i* + 1)th source, (c) a region illuminated by both the (*i*)th and the (*i* + 1)th source, and (d) a region without illumination. The basic idea of the cone-angle dependent weighting is that, for a given view angle, each voxel in the reconstructed volume can select one longitudinal source to be back-projected (binary weighting), as in regions (a) and (b) in Figure 8 or choose multiple longitudinally offset sources with complementary (nonbinary) weights that depend on the cone-angle associated with each source location, like in region (c) in Figure 8. 

The actual implementation of the cone-angle dependent weighting approach is a bit more complicated, first because some voxels will not be illuminated from any source at all, as in region (d) in Figure 8, and second because sudden transitions from one source to the other will result in a discontinuity in the weights, which may introduce artifacts. 

To minimize discontinuities along different regions of reconstructed volume and still achieve cone-angle dependent weighting to effectively combine multi-axial acquisition data, feathering is required for smooth transition. The weight *w*_*cw*_(*r*, *z*, *i*) associated with voxel *x*(*r*, *z*) and the (*i*)th longitudinal source will be determined by the cone-angle of that voxel and satisfies

(6) $$\overset{N}{\underset{i=1}{\sum }}w_{cw}\left(r,z,i\right)=\overset{N}{\underset{i=1}{\sum }}w_{cw}\left(x\left(r,z\right),i\right)=1,$$

where *N* is the total number of longitudinally offset sources. The final reconstructed volume will be

(7) $$\hat{f}\left(\underline{x}\right)=\sum_{i=1}^{N}{\hat{f}}_{i}\left(\underline{x}\right),$$

where

(8) $$\hat{f_{i}}\left(\underline{x}\right)=\frac{1}{2_{\pi }}\int_{\Lambda_{h}}^{}\frac{w_{cw}\left(r,z,i\right)∙w_{h}\left(\lambda \;,\alpha \right)}{{\left|\underline{x}-\underline{\alpha }\left(\lambda \;,i\right)\right|}^{2}}\cdot p\left(\lambda ,\alpha ,\gamma ,i\right)*q\left(\sin\;\gamma \right)d\lambda ,$$

and *w*_*h*_(*λ*, *α*) is a half-scan view weighting factor, such as Parker weighting [20]. The weight *w*_*cw*_(*r*, *z*, *i*) should be continuous even though *x*(*r*, *z*) travels through the different regions shown in Figure 8. For example, the weight *w*_*cw*_(*r*, *z*, *i*) in region (a) should be 1 because only the (*i*)th source illuminates region (a). However, the weight in region (b) should be 0 because only the (*i* + 1)th source illuminates region (b). As a result, the weight changes abruptly at the boundary between region (a) and (b), and this discontinuity in weight will result in image artifacts. Therefore, we should force the continuity of the weights, *w*_*cw*_(*r*, *z*, *i*) spatially, either longitudinally or trans-axially (plane-wise). It turns out that the reconstruction is more sensitive to transaxial discontinuities in the weights.

Each region in Figure 8 can be assigned with weights depending on the data availability. In region (a), only illuminated by a given source, the weight is one. In region (b), not illuminated by that same source, the weight is zero. In region (c), illuminated by that same source and by other source, the weight is determined based on the respective cone-angles at which those two sources illuminate any given voxel. In region (c), close to the center plane of the (*i*)th source the weight approaches to one, and close to the edge of detector the weight approaches to zero. Region (d), where no illumination comes from any source, is a special case because no direct measurements are available. While we focus on computing weights by considering contributions from two adjacent sources, this method can be extended to have contributions from more than two sources. If longitudinal separation between the sources is small, some region will get illuminated by more than two sources.

**Figure 9:**
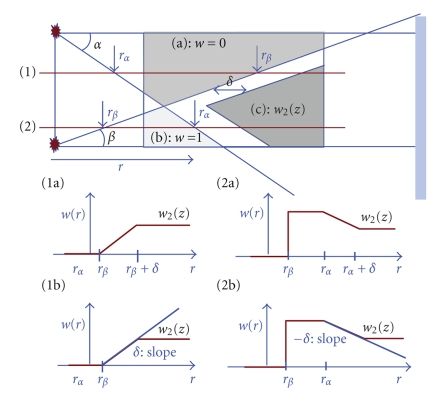
The top picture represents a longitudinal view of the proposed weighting scheme. Two specific slices are selected: slice (1) includes voxels from region (a) and region (c), and slice (2) includes voxels from region (b) and region (c). The corresponding weight profiles are shown at the bottom: (1a) and (2a) show the fixed feathering width case, where the width is defined as *δ*; (1b) and (2b) show the fixed feathering slope case, where the slope is defined as *δ*.

To make a smooth transition from region (a) to region (c) and from region (b) to region (c), we propose two approaches: (i) an approach with fixed feathering width and (ii) an approach with fixed slope but variable feathering width. We present a specific example in Figure 9. Slice (1) includes voxels *x*(*r*, *z*) from region (a) and region (c). As *r* increases, we move from region (a) to region (c), and the corresponding weight would abruptly change from 0 to *w*_2_(*z*), see (Figure 9 (1a)). A fixed feathering width *δ* is used to minimize the sudden weight change from *w*_start_(*z*) to *w*_final_(*z*). In this specific example, *w*_start_(*z*) = 0, and *w*_final_(*z*) = *w*_2_(*z*). Similarly, slice (2) includes voxels *x*(*r*, *z*) from region (b) and region (c). As *r* increases we move from region (b) to region (c), and the corresponding weight would abruptly change from 1 to *w*_2_(*z*) (Figure 9) (2a). In this case *w*_start_(*z*) = 1, and *w*_final_(*z*) = *w*_2_(*z*). Generally we define the following weighting scheme:

(9) $$w\left(r,z\right)=\left(\frac{w_{\text{final}}\left(z\right)-w_{\text{start}}\left(z\right)}{\delta }\right)\left(r-r_{\text{start}}\right)+w_{\text{start}},$$

where *δ* is defined as the (fixed) width of the transition region, see (Figures 9 (1a) and 9 (2a)). One drawback of this approach is that the difference between *w*_start_(*z*) and *w*_final_(*z*) can be very large at some locations, and the weight change will still be very abrupt. Another possible approach is to change the transition width such that we have a fixed transition rate or slope:

(10) $$w\left(r,z\right)=\delta \cdot \left(r-r_{\text{start}}\right)+w_{\text{start}},$$

where *δ* is defined as the transition rate, see (Figures 9 (1b) and 9 (2b)). 

Region (d) is treated separately because there is no ray passing through from any source. In this region, we extrapolate the detector data and make the weight *w*_1_(*z*, *r*) gradually change from 0 at the boundary with region (a) to 1 at the boundary with region (b). The equiweight lines where *w*_1_(*z*, *r*) is constant will form a fan focused at the corner where regions (a) and (b) meet. The contributions from extrapolated data by the (*i*)th source gradually fade away toward the (*i* + 1)th source and eventually reduce to 0. The size of region (d) is determined by system geometry parameters. For most of our multi-axial geometries described in Section 5, region (d) does not exceed 8% of the 50 cm scan field of view (FOV), and it does not overlap with the 35 cm cardiac FOV. 

With a limited number of longitudinal samples, this approach can still result in transaxial discontinuities. A simple way to avoid this is to oversample in *z*, compute the weights, and then average the weights in *z*. This approach is sensitive to the sampling on transaxial plane and longitudinal direction (slices). Note that the above weighting methods only provide continuity within transaxial planes and do not guarantee continuity along the longitudinal direction. Furthermore, the proposed weighting approach for half-scan mode can be applied to full-scan mode with utilizing whole detector, but resulting configuration will be less optimal in dose efficiency.

## 5. Numerical Results

**Figure 10:**
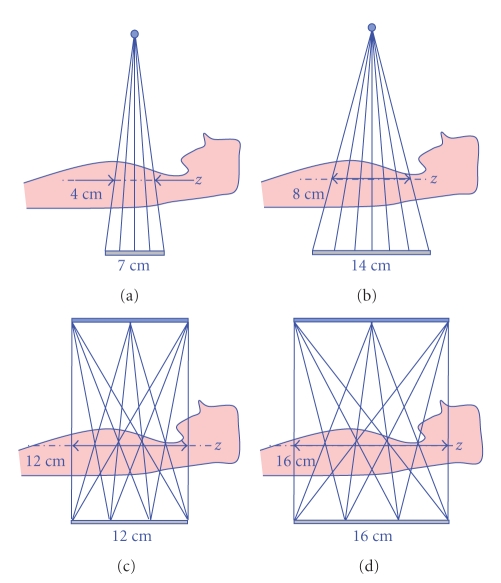
Illustration of proposed CT geometries: (a) and (b) are 3rd generation CT systems with 40 mm and 80 mm coverage, and (c) and (d) are multi-axial acquisition CT systems with 120 mm and 160 mm coverage. Note that the coverage of a multi-axial acquisition system is the same as detector size. 3rd generation system requires bigger detector for the same scan coverage.

To evaluate the proposed algorithms for multi-axial acquisitions, two conventional 3rd generation CT geometries and two multi-axial acquisition geometries are investigated, as presented in Figure 10: (a) a single axial acquisition with 40 mm scan coverage and detector height 70 mm; (b) a single axial acquisition with 80 mm scan coverage and detector height 140 mm; (c) three axial acquisitions separated by 60 mm, with 120 mm coverage and detector height 120 mm; (d) three axial acquisitions separated by 80 mm, with 160 mm coverage and detector height 160 mm. All four proposed geometries have the source-focused curved detector with same detector pitch (1 mm by 1 mm), the same number of detector columns (1024), and the same number of views (1024). We oversampled the detector rows, columns, focal spot, and rotation by a factor two to accurately model the X-ray beam width and azimuthal blur. We used a realistic CT simulation environment CATSIM [21] and a helical body phantom (HBP), designed to explore cone-beam artifacts [22]. We added additional nonparallel elliptical cylinders to the original HBP to better model the ribs. All acquisitions were simulated with 120 kVp polychromatic X-ray spectrum. 

Note that for 3rd generation CT system the scan coverage is defined as the projection of the physical detector to isocenter. On the other hand, the scan coverage for the studied multi-axial acquisition system is equal to the size of detector, which means that more coverage is obtained with the same size of detector, as shown in Figure 10. Furthermore, it is conceptually straightforward to increase the scan coverage of multi-axial acquisition system by combining more axial scans or by using more longitudinally offset sources. Also, the coverage can be adjusted by changing the spacing between the multiple axial scans, but cone-beam artifacts may become more severe. Similarly the detector can be extended, and the X-ray beam collimator opened up accordingly, again at the possible expense of cone-beam artifacts.

**Figure 11:**
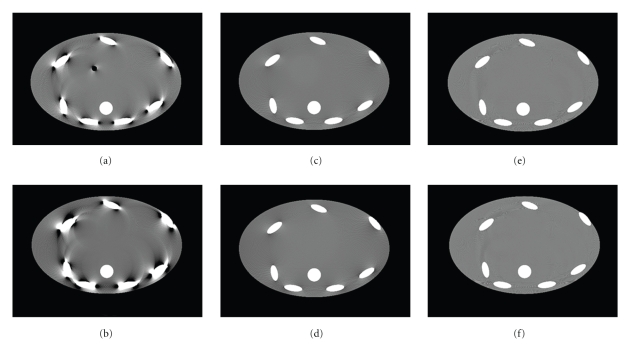
Worst-case images from (a) 3rd generation CT system with 40 mm coverage, (b) 3rd generation CT system with 80 mm coverage, reconstructed with FDK, (c) multi-axial acquisition CT system with 120 mm coverage, (d) multi-axial acquisition CT system with 160 mm coverage, reconstructed with slice substitution approach, using FDK slices, (e) multi-axial acquisition CT system with 120 mm coverage, and (f) multi-axial acquisition CT system with 160 mm coverage, reconstructed with TOM window-based reconstruction. Grayscale: (−50 HU, 50 HU). Note that the worst-case slices for 3rd generation system are the slices located at the edge of scan FOV, and the worst-case slices for multi-axial acquisition system are the slices located in between two sources.

Figures 11(a)  and 11(b)  show the worst-case slices for a full-scan 3rd generation geometry with 40 mm and 80 mm scan coverage, respectively. The images were obtained with standard FDK reconstruction in 1 mm by 1.88 mm voxel grid (pixel by slice thickness). The worst-case slices are located at the edge of the scan coverage because those slices see the largest cone-angle and are reconstructed with truncated data. Figures 11(c)  and 11(d)  show the worst-case slices for a full-scan multi-axial acquisition CT with 120 mm and 160 mm scan coverage. Here the images were reconstructed with the FDK-based slice substitution approach described in Section 2. The worst-case slices for the FDK-based slice substitution approach are where the adjacent slices come from different reconstruction volume. Figures 11(e)  and 11(f)  show the worst-case slices for a full-scan multi-axial acquisition CT with 120 mm and 160 mm scan coverage. Here the images were reconstructed with TOM windowing approach described in Section 3. The worst-case slices for the TOM windowing approach are halfway between two consecutive sources.

All images are displayed in tight window, (−50 HU, 50 HU). The results show that the cone-beam artifacts are very severe in the 3rd generation CT geometry. Images from FDK-based slice substitution approach show some residual cone-beam artifact (Figures 11(c)  and 11(d)), and the image from TOM windowing shows good image quality with very limited cone-beam artifacts (Figures 11(e)  and 11(f)). In all cases cone-beam artifacts increase with coverage. All six cases have slight view aliasing artifacts far from the isocenter due to imperfect oversampling in the simulation step. Note that the FDK-based slice substitution approach and TOM windowing approach show comparable image quality, but the TOM windowing approach has better dose efficiency than FDK-based approach. 

Figures 11(d)  and 11(f)  show the worst-case images from three axial acquisitions with 160 mm scan coverage and three axial acquisitions separated by 80 mm. 160 mm scan coverage is sufficient to cover most cardiac and head exams in a single rotation. As the spacing between adjacent sources gets larger, even larger scan coverage can be achieved, but at the expense of increased cone-beam artifact. Indeed, there are slightly increased shading artifacts in Figure 11(f) compared to Figure 11(e). Nevertheless, the cone-beam artifacts in 120 mm and 160 mm coverage multi-axial acquisitions are much less severe than those from 3rd generation CT system with smaller scan coverage, that is, 40 mm and 80 mm. In previous work we have demonstrated extended scan coverage up to 200 mm [16, 23].

**Figure 12:**
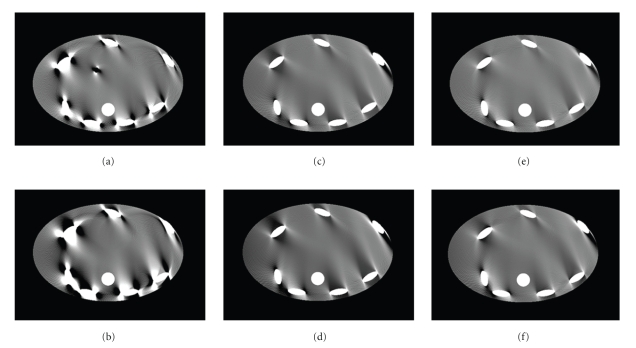
Worst-case images from (a) 3rd generation CT system with 40 mm coverage, (b) 3rd generation CT system with 80 mm coverage, reconstructed using FDK with Parker weighting approach, (c) multi-axial acquisition CT system with 120 mm coverage, (d) multi-axial acquisition CT system with 160 mm coverage, reconstructed with slice substitution approach using FDK parker weighting slices, (e) multi-axial acquisition CT system with 120 mm coverage, and (f) multi-axial acquisition CT system with 160 mm coverage, reconstructed with cone-angle dependent weighting reconstruction. Grayscale: (−50 HU, 50 HU). Note that the worst-case slices for 3rd generation system are the slices located at the edge of scan FOV, and the worst-case slices for multi-axial acquisition system are the slices located in between two sources.

Figures 12(a)  and 12(b)  show the worst-case slices for a half-scan 3rd generation geometry with 40 mm and 80 mm scan coverage, respectively. The images were obtained with standard FDK reconstruction with Parker weighting in 1 mm by 1.88 mm voxel grid (pixel by slice thickness). Figures 12(c)  and 12(d)  show the worst-case slices for a half-scan multi-axial acquisition CT with 120 mm and 160 mm scan coverage. Here the images were reconstructed with FDK-Parker weighting-based slice substitution approach presented in Section 2. Figures 12(c)  and 12(d)  show the worst-case slices for a half-scan multi-axial acquisition CT with 120 mm and 160 mm scan coverage. Here the images were reconstructed with the cone-angle-based weighting approach presented in Section 4. 

In half-scan mode, the cone-beam data is always much less complete than in full-scan mode, specifically because conjugate rays are not available, and hence image artifacts are also much more severe. Figures 12(a)  and 12(b)  show various degrees of half-scan image artifacts for the 3rd generation CT system. Figures 12(c), 12(d), 12(e), and 12(f)  still show half-scan artifacts but strongly reduced compared to the 3rd generation case with much smaller coverage. Furthermore, the proposed cone-angle-based weighting approach, shown in Figures 12(e)  and 12(f), improves image quality even further from the FDK-Parker weighting-based slice substitution approach. Note that the region (d) in Figure 8 where no measurements are available gets bigger as the spacing between consecutive sources gets larger. For the full-scan case, larger scan coverage can be achieved at the expense of increased cone-beam artifact, but for the half-scan case, larger scan coverage is not only limited by the increasing cone-beam artifact but also the increasing missing data region.

Since TOM windowing approach is based on the FDK type of implementation, additional correction approach such as radon space-based correction [24] might improve image quality further, which may be a topic of future investigation.

## 6. Conclusion

In this paper, we present multi-axial CT acquisition geometries, which can be implemented by performing multiple axial scans with a single source 3rd generation system or by performing one or more axial scans with a multi-source CT system, in which sources are offset longitudinally. We propose corresponding reconstruction algorithms for full-scan and half-scan protocols. Both the TOM windowing reconstruction algorithm for full-scan mode and the cone-angle dependent weighting reconstruction algorithm for half-scan mode successfully reduce cone-beam artifact compared to a 3rd-generation CT acquisition with a single circular trajectory, even when the scan coverage is increased up to 160 mm. The same techniques can be applied to an inverse-geometry CT system.

Multi-axial CT geometries offer additional benefits associated with the reduced cone-angle, such as reduced Heel effect and reduced scatter, and there is room to optimize the target angles for each of the longitudinally offset spots. We have published some work in this area in [25], but those topics are beyond the scope of this paper.
